# Sleepiness as a Local Phenomenon

**DOI:** 10.3389/fnins.2019.01086

**Published:** 2019-10-18

**Authors:** Sasha D’Ambrosio, Anna Castelnovo, Ottavia Guglielmi, Lino Nobili, Simone Sarasso, Sergio Garbarino

**Affiliations:** ^1^Dipartimento di Scienze Biomediche e Cliniche “L. Sacco”, Università Degli Studi di Milano, Milan, Italy; ^2^Sleep and Epilepsy Center, Neurocenter of Southern Switzerland, Civic Hospital (EOC) of Lugano, Lugano, Switzerland; ^3^Department of Neuroscience, Rehabilitation, Ophthalmology, Genetics and Maternal/Child Sciences, University of Genoa, Genoa, Italy; ^4^Department of Neuroscience (DINOGMI), University of Genoa, Genoa, Italy; ^5^IRCCS, Child Neuropsychiatry Unit, Giannina Gaslini Institute, Genoa, Italy

**Keywords:** local sleep, sleepiness, microsleep, EEG slowing, sleep loss, prolonged wakefulness, OFF-periods, performance

## Abstract

Sleep occupies a third of our life and is a primary need for all animal species studied so far. Nonetheless, chronic sleep restriction is a growing source of morbidity and mortality in both developed and developing countries. Sleep loss is associated with the subjective feeling of sleepiness and with decreased performance, as well as with detrimental effects on general health, cognition, and emotions. The ideas that small brain areas can be asleep while the rest of the brain is awake and that local sleep may account for at least some of the cognitive and behavioral manifestations of sleepiness are making their way into the scientific community. We herein clarify the different ways sleep can intrude into wakefulness, summarize recent scientific advances in the field, and offer some hypotheses that help framing sleepiness as a local phenomenon.

## Introduction

Epidemiological data have shown that, over the last decades, we are seeing a concerning decrease in both the duration and the quality of sleep in developed and developing countries (Dinges, 1995; Broman et al., 1996; Dinges et al., 1997; Liu and Zhou, 2002; Krueger and Friedman, 2009; Maric et al., 2017). The progressive shift toward “24-h societies” has been accompanied by an increase in “sleepiness” and its associated detrimental effects on the individual’s performance, cognition, emotions, and general health (Dinges et al., 1997; Van Dongen et al., 2003a; Chee and Choo, 2004; Banks and Dinges, 2007; Bernert and Joiner, 2007; Knutson et al., 2007; Goel et al., 2009, 2014; Couyoumdjian et al., 2010; Grandner et al., 2010; Krause et al., 2017). Thus, understanding the regulatory mechanisms of sleepiness and their implications for human health is urgent and of utmost importance (Garbarino et al., 2016).

Although the concept of sleepiness might sound intuitive, at a closer look its definition is far from trivial, and neither is the answer to fundamental issues like what sleepiness is from a neurobiological standpoint. Attempts to operationalize the subjective feeling of sleepiness for clinical and research purposes have led to the development of a number of tools, some based on subjective ratings (e.g., the Epworth Sleepiness Scale), others on objective measures like cognitive performance (e.g., reaction time test, driving-simulators) and electroencephalography [e.g., multiple sleep latency test (MSLT) or polysomnography (PSG)]. Despite the reliability of these validated measures, their agreement remains poor as they capture different aspects of sleepiness, differentially influenced by endogenous, exogenous, and situational factors. For tackling and overcoming the complex phenomenon of sleepiness, previous studies have employed and suggested a twofold approach of identifying “sleepy” patients based on combined subjective and objective sleepiness and/or physiological and biochemical biomarkers (Olson et al., 1998; Kritikou et al., 2014); this approach may be more valuable than any single measure of sleepiness (Fleming et al., 2016) but is not yet exhaustive.

In the last few years, science has produced compelling evidence supporting the idea that both sleep and wakefulness are under local regulation (Siclari and Tononi, 2017; Krueger et al., 2019). These ideas were influenced by studies performed during the transition between wake and sleep, where it was found that some brain areas may fall asleep, or awaken, before others (Pigarev et al., 1997; Magnin et al., 2010; Marzano et al., 2013; Sarasso et al., 2014a, b; Siclari et al., 2014). In further support of this view, sleep homeostasis can be modulated on a local level by active or passive tasks or via local synaptic potentiation (Kattler et al., 1994; Miyamoto et al., 2003; Huber et al., 2004, 2006, 2007; De Gennaro et al., 2008; Vyazovskiy and Tobler, 2008; Hanlon et al., 2009; Lesku et al., 2011).

The presence of local sleep was also demonstrated through the observation that slow waves and spindles, the two major spontaneous electroencephalographic oscillations of sleep that arise from complex re-entrant circuits in the thalamocortical system, often occur out-of-phase in different brain regions (Andrillon et al., 2011; Nir et al., 2011).

This case is particularly compelling as both sleep spindles and slow waves are dependent on the hyperpolarization of thalamic relay and cortical neurons, respectively. This occurs during NREM sleep due to the progressive decrease of noradrenergic, serotonergic, and cholinergic neuromodulation from brainstem activating systems. As such, being under the influence of diffuse neuromodulatory systems, their occurrence, particularly for slow waves, has been long assumed to be an ubiquitous feature of virtually every neural cell of the sleeping brain (Steriade et al., 1993) occurring in a remarkably synchronous way (Volgushev et al., 2006).

Even more dramatically, during non-rapid eye movement (NREM) sleep, slow waves may coexist with transient local wake-like activity (Rector et al., 2005, 2009; Nobili et al., 2011; Peter-Derex et al., 2015). Similarly, local isles of sleep may intrude upon wakefulness (Rector et al., 2005, 2009; Vyazovskiy et al., 2011a; Hung et al., 2013; Quercia et al., 2018).

We herein clarify the different ways sleep can physiologically intrude into wakefulness, summarize the main findings on this topic, and offer a global framework to interpret sleepiness as a local phenomenon.

## The Intrusion of Sleep Into Wakefulness

From a neuro-physiological standpoint, sleep may intrude upon wakefulness in the form of local sleep, electroencephalogram (EEG) slowing, and microsleep.

### Local Sleep During Wakefulness

Local sleep is a complex physiological phenomenon occurring within anatomically discrete brain locations (Krueger et al., 2019). Experiments in isolated cortical slabs (Kristiansen and Courtois, 1949), as well as in slice preparations (Steriade et al., 1993) and cell cultures (Corner et al., 2008; Hinard et al., 2012), confirmed that slow waves—a key electrophysiological graphoelement characterizing the sleep state—is essentially an intrinsic property of cortical cells ensembles. Rector et al. (2005) provided the first indirect evidence of local sleep in living intact animals using surface evoked potentials (SEP) in rats. They showed that, while on average, wakefulness is characterized by low SEP amplitude and NREM sleep by high SEP amplitude (Hall and Borbely, 1970), SEP amplitude fluctuates over time during both states and is frequently different between hemispheres and nearby cortical columns. Moreover, the longer a cortical column produces low-amplitude wake-like SEP the more it will begin to produce large-amplitude sleep-like SEP (Rector et al., 2005).

Further indirect evidence in favor of local sleep emerged from cortical multiunit recordings in rats during sleep deprivation (SD): firing rates increased continuously for the first 3 h of SD and showed no further significant change in the last hour. This “ceiling-effect” was interpreted as the consequence of the increase in the number of local neuronal silent periods (or OFF-periods) (Vyazovskiy et al., 2009).

Local sleep had been observed more directly in rats in 2011 (Vyazovskiy et al., 2011a, b). After a period of prolonged wakefulness, cortical neurons tended to fall silent for brief periods, as they do during NREM sleep. These OFF-periods were associated with slow oscillations in the slow/theta range in local field potential (LFP) recordings, as also confirmed by a study applying micro-stimulation during prolonged wakefulness (Vyazovskiy et al., 2013). Local OFF-periods occurred asynchronously across brain regions and increased with time spent awake. Most strikingly, they occurred in behaviorally awake animals, and their presence over motor areas negatively affected motor performance during a sugar pellet reaching task.

More recently, use-dependent, local sleep-like EEG theta events have been found to occur during prolonged wakefulness in humans (Hung et al., 2013). As in the former studies on rodents, also in humans the occurrence of these events over frontal or posterior scalp regions was selectively associated with negative behavioral outcomes on executive functions or visuomotor control tasks, respectively (Bernardi et al., 2015; Quercia et al., 2018).

These findings lead to the intriguing hypothesis that deficits in sensory, psychomotor, and cognitive aspects of behavior after SD may arise as a result of altered neuronal responsiveness to incoming stimuli due to these OFF-periods.

### Global and Local EEG Slowing

Under SD conditions, which are similar to what occurs during NREM sleep, the firing rates of neurons in ON-periods, as well as the number and duration of OFF-periods, the number of neurons participating synchronously in OFF-periods and the low-frequency content (particularly in the theta range) of the EEG increase (Vyazovskiy et al., 2011b). At odds with NREM sleep, though, is the fact that during SD these events typically involve an isolated portion of the cortex possibly reflecting the occurrence of more or less widespread local cortical isles of sleep during prolonged wakefulness. In further support of this hypothesis, an fMRI connectivity analysis indicated that prolonged wakefulness is associated with a decrease in measures representing the mean strength of coupling among brain areas, resembling the breakdown in connectivity typical of slow waves sleep (Bernardi et al., 2015; Kaufmann et al., 2016).

In humans (Cajochen et al., 1995; Aeschbach et al., 1997; Finelli et al., 2000; Strijkstra et al., 2003; Fattinger et al., 2017), as in rats (Franken et al., 1991; Vyazovskiy and Tobler, 2005), EEG power in the lower frequency bands (especially theta) progressively increase with the time spent in quiet waking. This slowing can be captured by surface EEG and is paralleled by subjective sleepiness—at least in humans (Aeschbach et al., 1996; Bernardi et al., 2015)—and by a decrease in behavioral performance (Gorgoni et al., 2014; Fattinger et al., 2017). Transcranial magnetic stimulation (TMS) measures converge with EEG measures in indicating that SD has severe effects on cortical activity. SD is associated with an increased TMS resting motor threshold and cortical facilitation, at least in females, and these changes clearly predict changes in EEG theta activity (De Gennaro et al., 2007).

The increase in low frequencies in the EEG is associated with the subsequent homeostatic increase of sleep slow-wave activity (SWA) during NREM sleep in both humans (Finelli et al., 2000) and rats (Borbély et al., 1984; Tobler and Borbely, 1986), as well as with the increase of theta and delta power during REM sleep (Tinguely et al., 2006). Notably, superimposed to the homeostatic process, several studies reported a strong circadian modulation of the waking EEG (Aeschbach et al., 1996; Dumont et al., 1999; Cajochen et al., 2002) so that the intrusion of low frequencies is restrained when sustained wakefulness coincides with the biological day, while it is completely free to manifest itself during the biological night (Cajochen et al., 2002). Similarly, cortical excitability assessed using TMS coupled with scalp EEG is robustly correlated with circadian dynamics and with endocrine markers of circadian amplitude (Ly et al., 2016).

Interestingly, these regulatory mechanisms may act locally. The aforementioned increase in low frequencies has a fronto-central predominance, mainly in the theta range during wakefulness (Finelli et al., 2000; Strijkstra et al., 2003) and in the SWA range during subsequent recovery sleep (Cajochen et al., 1999; Finelli et al., 2000; Leemburg et al., 2010), and it can be observed also after repeated partial sleep restriction (Plante et al., 2016).

More recently, studies performed in humans showed that the increase in waking theta EEG activity during SD displayed regional, use-dependent changes (Hung et al., 2013; Gorgoni et al., 2014; Bernardi et al., 2015; Nir et al., 2017). A first study took advantage of high-density EEG technology to show an increase in theta power over left frontal brain regions after a language-based task and over posterior parietal regions after a visuomotor task. The same regions displayed local increases in SWA power during subsequent recovery sleep (Hung et al., 2013). A subsequent study demonstrated that the occurrence of theta waves in task-related regions coincided with specific performance errors in humans (Bernardi et al., 2015). Another study used intra-cranial electrodes in human neurosurgical patients performing a psychomotor vigilance task (PVT) at baseline and during SD. Cognitive lapses involved local state-dependent changes in neuronal activity in the medial temporal lobe (MTL). Specifically, immediately before cognitive lapses the spiking responses of individual neurons were attenuated, delayed, and lengthened while, during cognitive lapses, LFPs showed a relative local increase in slow activity (Nir et al., 2017).

In line with these findings, a study using a driving simulator to evaluate the effect of sleepiness at the wheel, found that a local increase in theta EEG activity over the motor regions (as localized by EEG source modeling techniques) was associated with an increased risk of line departures (Ahlstrom et al., 2017).

### Microsleep

In this review, microsleeps are defined as short episodes of sleep-like activity that satisfy criteria for stage 1 sleep (theta replacing alpha rhythm) except for their short duration of up to 15 s (Priest et al., 2001; Blaivas et al., 2007; Hertig-Godeschalk et al., 2019). Usually, blinking artifacts characteristic of full wakefulness disappear, often accompanied by the appearance of slow eye movements. However, behavioral changes, such as eye-closure and nodding-off, are not defining features of microsleeps, as they may or may not be present during microsleep episodes (Torsvall and Akerstedt, 1988; Boyle et al., 2008). Regardless, microsleeps may be associated with significant cognitive impairment—e.g., poorer performance during a continuous task under driving-simulator conditions (Boyle et al., 2008)—and are strictly associated with subjective sleepiness. Indeed, some evidence suggests that microsleeps analysis in MSLT might be a more sensitive and specific test for excessive daytime sleepiness (EDS) as compared to MSLT alone (Tirunahari et al., 2003).

Microsleep episodes are more frequent after a sleep-restricted night compared to a normally rested night (Friedman et al., 1978; Horne and Pettitt, 1985; Poudel et al., 2018) and can be followed by a brief recovery in performance (Poudel et al., 2018).

Traditionally, microsleeps have been hypothesized to be global brain phenomena that reflect the transient shutdown of activating systems, with the parallel activation of sleep promoting centers (Sechenova, 2011; Silkis, 2013). However, recent evidence describes microsleep in terms of intermediate states between sleep and wakefulness (Hertig-Godeschalk et al., 2019), possibly reflecting their local nature. Supporting this notion, a recent fMRI study during one night of SD described local decreased activation over frontal, parietal, and occipital associative cortices as well as increased activation in the default mode network (DMN) associated with slow reaction times responses at the PVT (typically reflecting the occurrence of microsleeps), showing how these different patterns of activation and deactivation could depend on circadian phases as well as homeostatic sleep pressure and the interactions between the two (Zhu et al., 2018).

## Neurobiology and Neurophysiology of Sleepiness

In simple terms, just as hunger is the physiological need for food, sleepiness can be described as the physiological need for sleep. Very few theoretical constructs about sleepiness are available in the literature (Cluydts et al., 2002; De Valck and Cluydts, 2003). Conceptually, sleepiness is the consequence of an imbalance between the sleep drive (level of activation of sleep circuits) and the wake drive (level of activation of arousal systems) (Cluydts et al., 2002). When wake still prevails but sleep pressure is high we experience sleepiness.

The concept of sleepiness, therefore, closely relates to the concept of “sleep debt.” While Horne originally proposed that sleep debt was uniquely the consequence of the loss of “core” or “obligatory sleep” (referred as the first 4–5 h of sleep) but not of “optional” or “facultative” sleep, which “fills the tedious hours of darkness until sunrise” (Horne, 1985), empirical research indicated that sleep debt accumulates linearly. Although clearly influenced and modulated by circadian factors (Shekleton et al., 2013), according to this line of research, sleep debt may be defined as the cumulative hours of sleep loss with respect to a subject-specific daily need for sleep (Van Dongen et al., 2003b).

Another closely related concept of sleepiness is “sleep inertia,” a physiological condition of subjective drowsiness, decreased alertness, and impaired cognitive and sensory-motor performance that arises during the transition between sleep to wakefulness. It has been shown that the subjective feeling of sleep inertia lasts on average 15–30 min, although objective measure of alertness and performance do not return to waking baseline until 2–4 h after waketime (Jewett et al., 1999). Electroencephalographic, evoked potential, and neuroimaging studies suggested that sleep inertia involves the intrusion of sleep patterns during wakefulness (Trotti, 2017), bringing the concepts of sleepiness and sleep inertia even closer and further corroborating the notion that vigilance states are not necessarily discrete.

But how does this translate from a neurophysiological standpoint? According to the data presented in the previous section, sleepiness may be associated with the occurrence of local sleep during wakefulness in the presence of a positive sleep debt. We will now focus on possible regulatory mechanisms of local sleep and their interaction with global processes.

### Local Regulation of Sleep

Great progress has been made in characterizing the brain centers responsible for the orchestration of sleep and wakefulness as global behavioral states (Jones, 2005; Saper et al., 2005; Szymusiak and McGinty, 2008; Siegel, 2009; Brown et al., 2012). Although anatomically widespread, these centers act in a coordinated fashion in modulating whole-brain activity, thus allowing for a clear behavioral distinction between wake and sleep states (for a review see Saper et al., 2001; Scammell et al., 2017; Eban-Rothschild et al., 2018). According to Saper’s flip-flop switch model, sleep regulation depends on a mutually inhibitory interaction between sleep centers and the components of the arousal systems (Saper et al., 2001), located both in cortical and subcortical structures.

Although the described mechanisms may orchestrate sleep globally, sleep is fundamentally an intrinsic property of the cerebral neurons and can be regulated locally at the level of cortical areas as small as cortical columns (Krueger et al., 2008). This columnar state segregation is favored by the fact that the functional intracolumnar connections are denser than intercolumnar connections allowing greater activity and state synchrony between cells pertaining to the same column (Panzeri et al., 2003).

The very first model-based postulation of local sleep was published in 1993 and 1995 by Krueger and Obal (1993) and Krueger et al. (2008), who postulated that sleep begins as a local neuronal group event involving oscillations of inhibition and excitation and is thus “quantal” in nature. From this perspective, these authors considered sleepiness as a statistical phenomenon, the perception of which arises when a sufficient number of neuronal groups become “bistable.”

Coordination of neuronal group sleep results from both neuronal and humoral systems. As proposed by the Synaptic Homeostasis Hypothesis (SHY; Tononi and Cirelli, 2003, 2006), when neuronal plasticity during wakefulness is increased or decreased in specific brain areas, sleep intensity, as reflected by the amount of SWA, selectively increases or decreases in those areas (Kattler et al., 1994; Huber et al., 2004; Vyazovskiy and Tobler, 2008; Hanlon et al., 2009; Lesku et al., 2011). Indeed, increased synaptic connectivity means more synchronous oscillations and increased slow wave activity. Alternatively (or additionally), local slow wave generation could be due to a change in the excitability or amount of adaptation of individual neurons. Along these lines, a build-up in the need for cellular maintenance could cause individual neurons to show lower excitability and stronger adaptation (Vyazovskiy et al., 2013). OFF-periods would therefore occur locally were most needed, thus providing a potential explanation for local sleep patterns. Similarly, as soon as individual neurons fall below a certain cellular stress threshold, their excitability is restored, leading to a more wake-like pattern of activity.

Considerable evidence also suggests a role for local paracrine signaling pathways in the regulation of both global and local sleep. In this vein, it is known that sleep pressure correlates with the concentration of—among others—nitric oxide (NO) (Gautier-Sauvigné et al., 2005; Kalinchuk et al., 2011), adenosine, and various cytokines (Imeri and Opp, 2009) such as interleukin-1 (IL-1) and tumor necrosis factor (TNF). These substances are synthesized by metabolically or synaptically active cells and are released in a local fashion (Latini and Pedata, 2001; Porkka-Heiskanen and Kalinchuk, 2011). Again, Krueger et al. (1995b) played a pivotal role in delineating this humoral component (Krueger, 2008). They hypothesized that sleep at the neuronal group level is regulated by paracrine substances whose production and catabolism rates are synaptic use-dependent (Krueger et al., 1995a).

According to their model, Adenosine 5′-γ-ThiotriPhosphate (ATP) released during neurotransmission and acting on purine P2 receptors induces the release of IL1 and TNF. These cytokines in turn act on neurons to change their intrinsic properties (Krueger, 2008), directly or indirectly, altering the production of neuroendocrine substances and neurotransmitters, for example the growth hormone releasing hormone and NO, which are known to be involved in sleep-wake regulation (Krueger et al., 1995b). More recent evidences in support of this model have been shown by Nguyen et al. (2019).

Finally, there is another mechanism able to explain regional sleep differences, especially the aforementioned frontal predominance of the low frequency effect. Different regions might be more susceptible to sleep due to intrinsic differences in some of their activating inputs. In other words, even widespread projections from centers regulating sleep globally may present with topographical differences that may affect sleep-wake regulation at the level of cortical macro-areas. For example, recent evidence suggested a region-specific dissociation between cortical noradrenaline levels during the sleep/wake cycle (Bellesi et al., 2016). Compared to the motor cortex, in the medial Prefrontal Cortex (mPFC) noradrenaline levels are higher and changes in its concentration during sleep and wake are slower. Furthermore, SD leads to a decrease in noradrenaline only at the level of mPFC, suggesting that noradrenergic neurons targeting the prefrontal cortex may undergo fatigue earlier or more markedly than other projecting cells from locus coeruleus. An increased susceptibility of noradrenergic projections to the frontal cortex might explain frontal cognitive executive function impairments associated with sleepiness (Jones and Harrison, 2001) and why the frontal lobes display more evident electrophysiological signs of deep sleep after prolonged wakefulness (Plante et al., 2016). These mechanisms may, alone or in combination, explain the occurrence of local sleep during wakefulness, leading to the subjective feeling of sleepiness.

As a last note, it is worth mentioning here that, despite the fact that we mainly focused on the cortex when we tried to explain the origins of local sleep, NREM sleep patterns *in vivo* emerge from the interplay between the cortex and the thalamus, more specifically the thalamic reticular nucleus (TRN). It has been reviewed recently how cellular and functional TRN heterogeneity may account for some features of local NREM sleep (Vantomme et al., 2019). By experimentally modulating the activation and firing of the TRN neurons, it was indeed possible to rapidly induce slow wave activity (Lewis et al., 2015) as well as sleep spindles (Fernandez et al., 2018) in spatially restricted regions of the cortex. However, at the current state of the art, it remains unclear how the TRN contributes in terms of physiological conditions and what the signals that activate TRN neurons locally are. Further research will need to clarify these aspects and disentangle the causative role—if there is one —of TRN and the cortex in this loop-network.

### Interplay Between Local and Global Regulation

Having highlighted local sleep regulatory mechanisms, it remains to be discussed how they interact with the different processes orchestrating sleep in a global fashion.

According to Saper’s model (Saper et al., 2005), sleep regulation relies on three main streams: the “homeostatic” (Porkka-Heiskanen et al., 1997, 2000; Huang et al., 2007, 2011, 2014), the “circadian” (Chou et al., 2003; Fuller et al., 2006), and the “cognitive/emotional” (Chou et al., 2002; Sakurai et al., 2005; Yoshida et al., 2006). Each drive can potentially act globally as well as locally. Aside from the well-known homeostatic local sleep modulation discussed above, there is evidence of regional modulation of brain circadian rhythmicity. This has been demonstrated by a recent fMRI study quantifying changes in brain responses to a sustained-attention task across the circadian cycle, during baseline wakefulness, SD, and after recovery sleep (Muto et al., 2016). Subcortical areas exhibited a dominant circadian modulation that closely followed the melatonin profile but had no significant influence on sleep debt. Cortical responses also showed significant circadian rhythmicity, the phase of which varied across brain regions, as well as a widespread negative influence exerted by sleep pressure. The mechanisms of this local modulation are unknown, although the authors suggested the potential role of clock gene expression. Intriguingly, an EEG study showed that circadian rhythms modulate the incidence amplitude, frequency, and slope of slow waves (the latter being the most accurate marker of synaptic strength), with a dominant effect on central and occipital areas (Lazar et al., 2015).

**FIGURE 1:**
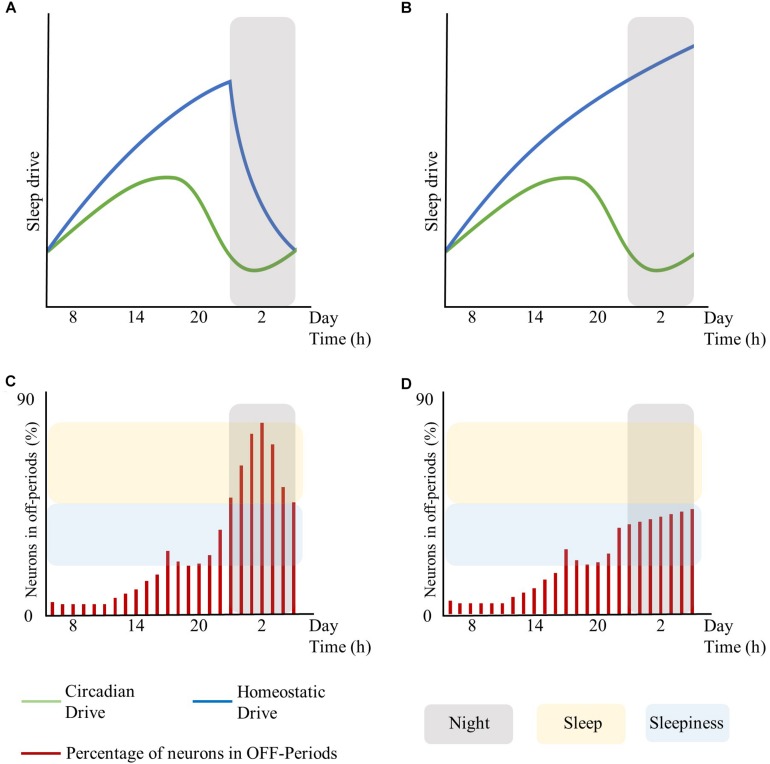
Interplay between global sleep drivers and cortical neuronal firing. The figure is intended to schematize the concepts described in this work without fitting any biological data for sleep drives or the number of neurons in OFF-periods. Top panels represent the time-course of the circadian and homeostatic drive over 24 h. Bottom panels represent the percentage of neurons in OFF-periods across 24 h. **(A)** circadian and homeostatic drives under physiological conditions. **(B)** circadian and homeostatic drives out of phase due to sustained wakefulness. **(C)** percentage of cortical neurons in OFF-periods under physiological conditions. **(D)** percentage of cortical neurons in OFF-periods during sustained wakefulness. Red bars: percentage of cortical neurons in OFF-periods. Blue lines: homeostatic drive. Green lines: circadian drive. Gray areas: night period. Yellow areas: sleep. Light-blue areas: sleepiness. The figure has been realized by fitting the mathematical function published in Daan et al. (1984).

Moreover, global regulatory mechanisms, particularly regarding the homeostatic and the circadian components, may influence local sleep regulation. In this respect, the extent of brain areas displaying sleep features (and thus the associated behavioral impairments and subjective feeling of sleepiness) may rest on the level of synchronization between global regulatory mechanisms. As such, asynchrony and shift of phase between the homeostatic and the circadian drive may result in local sleep without a global state transition (see Figure 1, conceived for schematizing these concepts without fitting any biological data for the sleep drives or for the number of neurons in OFF-periods). Likewise, the cognitive/emotional system may modulate the interaction between the homeostatic and the circadian drives and keep the subject awake despite strong circadian and homeostatic sleep-promoting inputs, accentuating their desynchronization (Horne, 1985).

In summary, local sleep may arise as an intrinsic property of each regulatory drive of the slow-wave cycle or by the desynchronization between these drives acting globally.

In turn, local sleep may affect brain centers responsible for sleep and wake as global behavioral states. As such, the occurrence of isolated local OFF-periods during wakefulness could subsequently lead to global sleep through the involvement of neuro-modulatory systems responsible for the generation of NREM sleep (Saper et al., 2010; Brown et al., 2012). Similar to focal onset seizures with impaired awareness, local changes in cortical activity may lead to profound global alteration of the vigilance state associated with loss of consciousness through the progressive involvement of other brain regions such as midline subcortical structures including the thalamus, the hypothalamus, and the brainstem (Englot and Blumenfeld, 2009). Recordings in the ventrolateral preoptic nucleus (VLPO) neurons showed that their firing rates increase during sleep, almost doubling during recovery sleep after SD, but did not increase during prolonged wakefulness. Thus, as homeostatic sleep drive accumulates, it may influence other neurons in the brain, such as the median preoptic neurons, which provide input to the VLPO (Saper et al., 2010). Alternatively, there could be a threshold in the number of areas showing sleep signs during wakefulness, which may imply behavioral impairments and sleepiness at first, and only when passing the threshold of the transition to global sleep. Specifically, it is hypothesized that whole organism sleep is an emergent property of the collective neuronal assemblies (Rector et al., 2009), as when networks of neuronal assemblies are coupled they will tend to synchronize (Roy et al., 2008). As such, when the number of neuronal groups entering the sleep state exceeds a significant threshold, other groups will follow (Rector et al., 2009) thus enabling the full-fledged transition from wakefulness to sleep (see Figure 1, conceived for schematizing these concepts without fitting any biological data for the sleep drives or number of neurons in OFF-periods).

Sleepiness typically arises in conditions of SD and/or prolonged wakefulness. The prevalence of the so called “insufficient sleep syndrome” is estimated to be between 1 and 4% of the population (Ohayon, 2008) and two to four times higher in individuals sleeping less than 6 h per night compared to individuals sleeping between 7 and 8 h per night (Ohayon, 2012).

The great majority of sleep disorders determine sleepiness through the curtailment of total sleep time and/or sleep fragmentation without primarily affecting the function of sleep-promoting centers. Examples are sleep breathing disorders like obstructive sleep apnoea, circadian rhythm sleep-wake disorders like shift-work disorder, sleep related movement disorders like restless leg syndrome, and objective insomnia. Central disorders of hypersomnolence like narcolepsy type 1 and 2 or idiopathic hypersomnia involve instead a pathologic imbalance between sleep promoting and wake promoting pathways in favor of the former, determining an increased sleep need and/or the abrupt intrusion of sleep into wakefulness due to an instability of the switching mechanisms between sleep and wakefulness. Also, parasomnias like sleepwalking and sleep terrors have been associated with subjective daytime sleepiness, via as of yet unknown mechanisms (Carrillo-Solano et al., 2016).

Sleepiness is also related to mental and organic diseases that may directly or indirectly affect sleep (Ohayon, 2012). These disorders may cause a disruption of the sleep-wake schedule due to changes in behavior, dysregulate sleep centers orchestrating sleep due to neurological lesions or more subtle abnormalities, or nociceptive, immunomodulatory, or other modulatory inputs.

## Conclusion and Future Directions

While familiar to all on a subjective level, sleepiness is a complex matter that science is just beginning to understand. We have herein summarized how, during prolonged wakefulness, the occurrence of local neuronal OFF-periods may relate to the well-known negative consequences on performance observed in this state. As suggested by the reviewed literature, this phenomenon of local sleep during wake may account for at least some of the cognitive and behavioral manifestations of sleepiness. Under this perspective, sleepiness may reflect the transition between different vigilance states, being an epiphenomenon of these “fluid boundaries” (Sarasso et al., 2014a).

This interpretation is probably the key that will help develop new measures to quantify sleepiness in the near future. From a clinical perspective, high-density EEG, which allows an optimal spatial and temporal resolution to capture local isles of EEG slowing, may represent a valuable technological support. Moreover, a better characterization of the role of the circadian rhythm and of its interaction with other drives that modulate the sleep-wake cycle is warranted. Finally, a promising future line of research will be on the linking of the neurophysiological concepts of local sleep and sleepiness to interindividual variability in susceptibility to sleepiness (Van Dongen et al., 2004; Kuna et al., 2012; Rupp et al., 2012; Goel et al., 2013; Spaeth et al., 2015). This will open the way to a more personalized sleep medicine that will have a considerable impact on human health and promote occupational well-being, benefiting the society as a whole.

## Author Contributions

AC and SD’A wrote the first draft of the manuscript. OG, SG, LN, and SS wrote sections of the manuscript. All authors contributed to the manuscript revision, read, and approved the submitted version of the manuscript.

## Conflict of Interest

The authors declare that the research was conducted in the absence of any commercial or financial relationships that could be construed as a potential conflict of interest.
